# 2D/3D Copper-Based Metal-Organic Frameworks for Electrochemical Detection of Hydrogen Peroxide

**DOI:** 10.3389/fchem.2021.743637

**Published:** 2021-10-07

**Authors:** Xiangjian Guo, Chuyan Lin, Minjun Zhang, Xuewei Duan, Xiangru Dong, Duanping Sun, Jianbin Pan, Tianhui You

**Affiliations:** ^1^ School of Nursing, Guangdong Provincial Key Laboratory of Pharmaceutical Bioactive Substances, Guangdong Pharmaceutical University, Guangzhou, China; ^2^ Center for Drug Research and Development, Guangdong Provincial Key Laboratory of Pharmaceutical Bioactive Substances, Guangdong Pharmaceutical University, Guangzhou, China; ^3^ State Key Laboratory of Analytical Chemistry for Life Science, School of Chemistry and Chemical Engineering, Nanjing University, Nanjing, China

**Keywords:** copper-based metal-organic frameworks, electrochemical sensor, hydrogen peroxide, dairy products, detection

## Abstract

Metal-organic frameworks (MOFs) have been extensively used as modified materials of electrochemical sensors in the food industry and agricultural system. In this work, two kinds of copper-based MOFs (Cu-MOFs) with a two dimensional (2D) sheet-like structure and three dimensional (3D) octahedral structure for H_2_O_2_ detection were synthesized and compared. The synthesized 2D and 3D Cu-MOFs were modified on the glassy carbon electrode to fabricate electrochemical sensors, respectively. The sensor with 3D Cu-MOF modification (HKUST-1/GCE) presented better electrocatalytic performance than the 2D Cu-MOF modified sensor in H_2_O_2_ reduction. Under optimal conditions, the prepared sensor displayed two wide linear ranges of 2 μM–3 mM and 3–25 mM and a low detection limit of 0.68 μM. In addition, the 3D Cu-MOF sensor exhibited good selectivity and stability. Furthermore, the prepared HKUST-1/GCE was used for the detection of H_2_O_2_ in milk samples with a high recovery rate, indicating great potential and applicability for the detection of substances in food samples. This work provides a convenient, practical, and low-cost route for analysis and extends the application range of MOFs in the food industry, agricultural and environmental systems, and even in the medical field.

## Introduction

Hydrogen peroxide (H_2_O_2_) is widely used in the food industry, medical field, textile industry, and paper industry (Zhang and Chen, 2017). Generally, H_2_O_2_ works as an antibacterial agent, bleaching agent (Kang et al., 2010), stabilizer, and preservative (Singh and Gandhi, 2015) in dairy products. Based on the laws and the rules, manufacturers are not allowed to add H_2_O_2_ in excess. H_2_O_2_ in abnormal level will damage human health, resulting in Alzheimer’s disease, cancer, and cardiovascular diseases (Upadhyay et al., 2014; Akyilmaz et al., 2017; Nascimento et al., 2017). Therefore, it is important to detect H_2_O_2_ in dairy products to protect public health and normalize the production with some benefits (Tang et al., 2010; Karimi et al., 2018). Nowadays, many analytical methods have been applied for the detection of H_2_O_2_, such as high-performance liquid chromatography (Ivanova et al., 2019), spectrophotometry (Li et al., 2017a), chemiluminescence (Li et al., 2017b), colorimetry (Dominguez-Henao et al., 2018; Yao et al., 2020), fluorescence (Pundir et al., 2018), and electrochemistry. Nevertheless, some of them are time consuming, of high consumption, and need advanced instruments or experienced and professional staff (Sun et al., 2016). Among them, electrochemistry has drawn attention due to rapid response (Ammam and Fransaer, 2010), high selectivity (Conzuelo et al., 2010), simple operation (Stankovic et al., 2020), and real-time detection. Electrochemical methods can be used as an alternative to other techniques as a result of their limited drawbacks. Dong et al. designed ZnFe_2_O_4_/SWCNTs/GCE as a new sensor for the electrochemical detection of pesticides in apples, tomatoes, leeks, and water samples (Dong et al., 2017). Vinitha Mariyappan et al. synthesized Gd_2_S_3_/RGO hybrid composites and modified on the surface of the glassy carbon electrode (GCE) to serve as an electrochemical platform for the detection of carbofuran in potatoes and river water samples (Mariyappan et al., 2021). Therefore, the electrochemical method is a promising strategy for the detection of H_2_O_2_ in dairy products.

A metal-organic framework (MOF) is a crystalline porous material constructed by coordination of metal ions or clusters with polytopic organic ligands (Furukawa et al., 2013). They possess many promising features like tunable structures, active sites, rapid electron transmission, and high surface area (Lee et al., 2009; Gu et al., 2014). MOFs have been extensively used in electrochemical applications (Chen et al., 2020; Lu et al., 2020; Wei et al., 2020), gas storages (Hinks et al., 2010; Zhang et al., 2020), and biomedical fields like wound healing (Fu et al., 2020; Chen et al., 2021), enhanced cancer therapy (Luo et al., 2019), imaging (Lu et al., 2018), antibacterial agents (Qi et al., 2020), cell detection (Shi et al., 2021), and drug delivery (Simon-Yarza et al., 2018) because of excellent physical and chemical properties. In addition, MOFs with catalytic activity have become an ideal modified material of electrochemical sensors for detection in real samples (Guo et al., 2020; He et al., 2020; Liu et al., 2020; Zhang et al., 2021). For example, Luan et al. prepared iron-based MOFs with modification (NMOF-Pt-sDNA) to detect kanamycin in milk samples (Luan et al., 2017). Zeng et al. modified copper-based metal-organic frameworks (Cu-MOFs) as a template to construct a nonenzyme electrochemical unit for H_2_O_2_ sensing in milk and human serum samples (Zeng et al., 2019). However, MOFs with different structures present a unique electrocatalytic property. Morphology and structure strongly affect their chemical and physical properties (Sun et al., 2020). Two-dimensional metal-organic frameworks (2D MOFs) with ultrathin thickness morphology and an ultrahigh surface area possess many accessible active sites on their surface. Thus, the catalytic and sensing applications could benefit from the inherent properties of 2D MOFs (Zhao et al., 2015; Zhao et al., 2018). Three-dimensional metal-organic frameworks (3D MOFs) with diverse morphology present outstanding chemical and physical properties in detection (Xue et al., 2019). It is meaningful to explore different structures of MOFs based on the same metal ions and study their electrochemical catalysis and other properties to investigate the mechanism.

As a typical series of MOFs, Cu-MOFs have been reported for many years. A classic version of 2D Cu-MOFs named Cu–TCPP has been successfully developed and applied in optoelectronic materials, catalysis, and sensing (Lu et al., 2016; Wang et al., 2016). Cu–TCPP has a large specific surface area, tunable pore size, 2D planar structure, and perfect nanostructure. Cu-TCPP is composed of Cu^2+^ as metal ions and tetrakis (4-carboxyphenyl) porphyrin (TCPP) as organic ligands (Dong et al., 2020). Porphyrin is a member of heterocyclic compounds with a conjugated structure. On the other hand, porphyrins are one of the substances with peroxide mimicking enzyme activity (Ma and Zheng, 2020). The surfactant, such as polyvinylpyrrolidone (PVP), plays a significant role in 2D MOF synthesis. On the one hand, the surfactant prevents the MOF layers from stacking in the vertical direction which is contributed to form ultrathin MOF nanosheets. On the other hand, PVP would maintain the as-synthesized MOF nanosheets in stabilization, preventing their aggregation (Zhao et al., 2018). According to previous reports (Bai et al., 2019; Ma et al., 2020), Cu-TCPP has been applied for the detection of H_2_O_2_ in real samples, showing the potential of fabricating electrochemical sensors to detect H_2_O_2_. One of the most representative Cu-MOFs with a 3D structure named HKUST-1 or MOF-199 was first reported and synthesized by the Hong Kong University of Science and Technology in 1999 (Chui et al., 1999). The main structural characterization of HKUST-1 is a copper dimer with a copper–copper distance of 0.263 nm. The material is composed of twelve oxygen atoms, obtained from the carboxylate groups of the four 1, 3, 5-benzenetricarboxylate (BTC) ligands, which are bound to the four coordination sites of each of the three Cu^2+^ ions. The presented paddle-wheel units form a face-centered crystal lattice with Fm-3m symmetry which possesses a three-dimensional porous network with a bimodal pore size distribution (Hartmann et al., 2008; Kim et al., 2012; Loera-Serna et al., 2012). It had an amount of open coordination sites, which was beneficial for detection (Li et al., 2018a). This kind of classic MOFs has been widely used in gas storage, biomedical field, and substance detection (Azad et al., 2016; Tan et al., 2017). However, there are little reports of pristine HKUST-1 as modified materials to construct an electrochemical sensor for the detection of H_2_O_2_ (Zhang et al., 2013; Yang et al., 2015). We are interested in investigating the comparison of 2D Cu-MOF (Cu-TCPP) and 3D Cu-MOF (HKUST-1) in H_2_O_2_ sensing.

**SCHEME 1:**
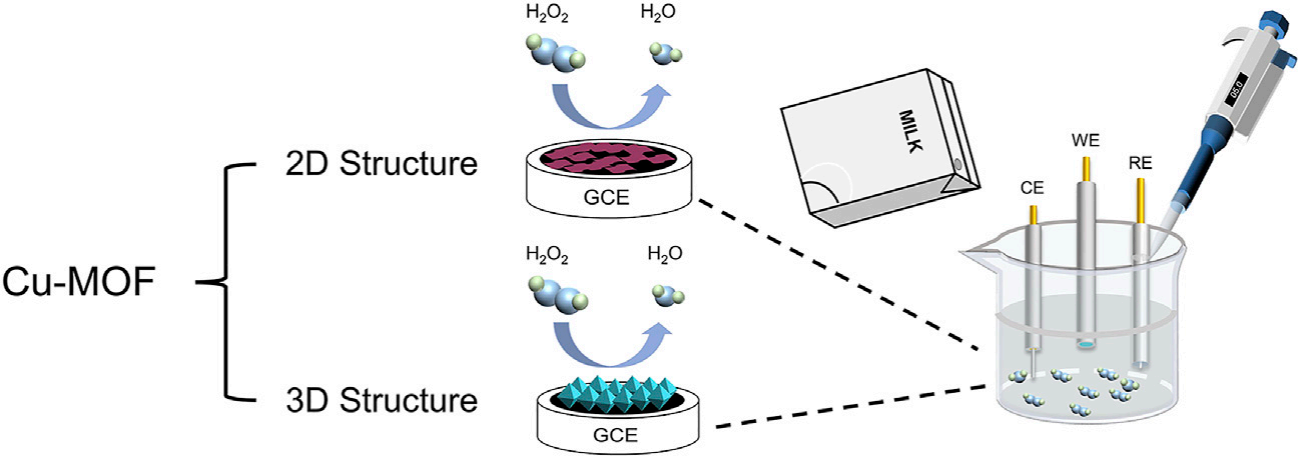
Schematic illustration of Cu-MOFs detecting H_2_O_2_ from the milk sample using the electrochemical method.

In this study, two kinds of different structures of Cu-MOFs were synthesized successfully. As shown in Scheme 1, the 2D Cu-MOF and 3D Cu-MOF were coated on the surface of GCE to construct electrochemical sensors, respectively. The HKUST-1/GCE displayed a better catalytic ability and electrochemical performance than Cu-TCPP/GCE in H_2_O_2_ reduction because of the three-dimensional structure and better conductivity. Besides, 3D Cu-MOF/GCE (HKUST-1/GCE) had two wide linear ranges of 2 μM–3 mM and 3–25 mM, and the limit of detection (LOD) was 0.68 μM with high sensitivity and selectivity. Based on these satisfactory results, the HKUST-1/GCE was successfully used for detecting H_2_O_2_ in milk samples. These results indicated the influences of structures and morphology of MOFs in electrochemical catalysis and made a great difference in the detection of substances. It pointed out the significance of investigating the morphology of MOFs for further exploring and studying the mechanism.

## Methods and Materials

### Materials and Reagents

Copper nitrate hydrate [Cu (NO_3_)_2_⋅xH_2_O], trimesic acid [C_6_H_3_(CO_2_H)_3_], sodium sulfate anhydrous (Na_2_SO_4_), citric acid monohydrate (C_6_H_8_O_7_⋅H_2_O), and ascorbic acid (C_6_H_8_O_6_) were obtained from Aladdin Reagent Co., Ltd. (Shanghai, China). Polyvinylpyrrolidone [PVP, molecular weight (Mw) = 40,000] was obtained from Sigma–Aldrich Co., Ltd. Tetrakis (4-carboxyphenyl) porphyrinabsolute (TCPP) was obtained from Tokyo Chemical Industry Co., Ltd. Sodium chloride (NaCl), disodium hydrogen phosphate (Na_2_HPO_4_), potassium chloride (KCl), potassium dihydrogen phosphate (KH_2_PO_4_), potassium ferricyanide [K_3_Fe(CN)_6_], and potassium ferrocyanide trihydrate [K_4_Fe(CN)_6_·3H_2_O] were obtained from Sinopharm Chemical Reagent Co., Ltd. (Shanghai, China). Ethanol absolute (EtOH) and N, N-dimethylformamide (DMF) were bought from Tianjin Damao Chemical Reagent Co., Ltd. Nafion (5%) was brought from Alfa Aesar Co., Ltd. Hydrogen peroxide (H_2_O_2_) and methanol (CH_4_O) were purchased from Guangzhou Chemical Reagent Co., Ltd. (Guangzhou, China). The phosphate buffered saline (PBS) (pH 7.2, 0.1 M) was prepared by mixing with 11.36 g Na_2_HPO_4_, 2.72 g KH_2_PO_4_, 0.20 g KCl, and 8.00 g NaCl into 1,000 ml ultrapure water. Ultrapure water (18.2 MΩ; Millipore Co., United States) was used to prepare all solutions. All solutions were stored at room temperature at 25 ± 2°C for further use. All reagents are of analytical grade without further purification.

### Apparatus and Instrumentation

Scanning electron microscopy (SEM) images were photographed by a scanning electron micrograph (SEM, Hitachi Regulus 8230, Japan). Transmission electron microscopy (TEM) images were taken by a transmission electron microscope (JEM 1400, Japan). Fourier transform infrared (FT-IR) spectra were conducted on a Fourier transformation infrared spectrometer (IR, EQUINOX 55, Germany). X-ray powder diffraction (XRD) patterns were recorded on a PANalytical instrument (Empyrean, Netherlands) to examine the crystal phase of the samples. The surface composition and valence states were studied by X-ray photoelectron spectra (XPS, Nexsa, Thermo Fisher Scientific, United States). All electrochemical experiments were studied by a CHI 660E electrochemical workstation (Shanghai CH Instruments Co., China). The traditional three-electrode system was employed in this research. The bare or modified glassy carbon electrodes, platinum electrode, and saturated Ag/AgCl electrode were served as working electrodes, counter electrodes, and reference electrodes, respectively.

### Synthesis of 2D Structure Cu-MOF

The synthesis process was based on a previous report (Ma and Zheng, 2020). First, 25 mg of Cu (NO_3_)_2_·xH_2_O and 100 mg PVP were dissolved in 60 ml solution containing DMF and Ethanol absolute (V: V = 3:1) under stirring condition. Second, 60 mg of TCPP was added to the above solution and further ultrasonicated. Finally, the solution was poured into a Teflon autoclave heating for 4 h using the solvothermal method at 80°C. The red product was centrifuged, washed, dried, and stored at room temperature. The red product was named Cu-TCPP or 2D Cu-MOF.

### Synthesis of 3D Structure Cu-MOF

The synthesis process was based on the preceding article (Wu et al., 2013). First, 1.82 g copper nitrate (Cu (NO_3_)_2_·xH_2_O) and 0.875 g trimesic acid (C_6_H_3_(COOH)_3_) were dissolved in 50 ml absolute methanol under ultrasonication to get blue and transparent solutions, respectively. Second, the copper nitrate solution was added to the trimesic acid solution. Third, the mixture solution was kept at room temperature for 2 h until 3D Cu-MOF precipitation was finished. The blue product was centrifuged and washed with methanol two times. Lastly, the blue product named HKUST-1or 3D Cu-MOF was dried in vacuum condition for use.

### Preparation of the Cu-MOF-Modified Electrode

Prior to modification, the bare GCE was polished with 0.05 mm Al_2_O_3_ powder and rinsed with deionized water and ethanol under ultrasonication for 2 min to get a mirror-like state. The mirror-like GCE was dried in nitrogen stream for use. 1 mg of 2D Cu-MOF or 3D Cu-MOF was dispersed in the solution containing ultrapure water and 5% Nafion solution (V: V = 2:0.004). 6 μL of 2D Cu-MOF or 3D Cu-MOF (1 mg/ml) dispersion was coated onto the surface of bare GCE and dried using an infrared lamp. The obtained electrodes are named Cu-TCPP/GCE and HKUST-1/GCE.

## Result

### Morphological, Structural, and Compositional Characterization of HKUST-1 and Cu-TCPP

**FIGURE 1:**
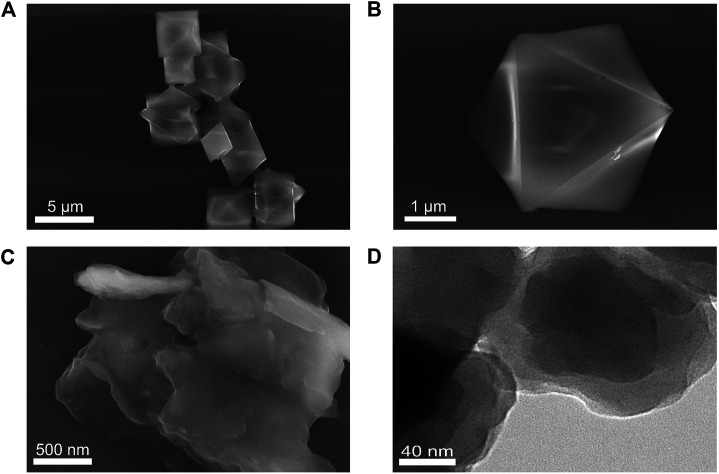
**(A)** SEM image of HKUST-1. **(B)** A magnified SEM image of HKUST-1. **(C)** SEM image of Cu-TCPP. **(D)** TEM image of Cu–TCPP.

The morphology, chemical composition, crystal structures, and functional groups of HKUST-1 and Cu-TCPP were characterized by scanning electron microscopy (SEM), transmission electron microscopy (TEM), X-ray photoelectron spectroscopy (XPS), powder X-ray diffraction (XRD), and Fourier transform infrared (FT-IR) spectroscopy. Figures 1A,B are the SEM images of HKUST-1. The prepared HKUST-1 displayed a uniform and octahedral structure with the size range of 1–3 μm. Figures 1C,D are the SEM image and TEM image of Cu-TCPP, respectively. The obtained Cu-TCPP displayed a two-dimensional and layer-by-layer structure with a wrinkled surface, indicating that the 2D Cu-TCPP nanosheets with an ultrathin structure had a large surface area. The two kinds of Cu-MOFs were consistent with the previously reported one (Wu et al., 2013; Ma and Zheng, 2020). Supplementary Figures S1A–D show the powders and solutions of Cu-MOFs.

**FIGURE 2:**
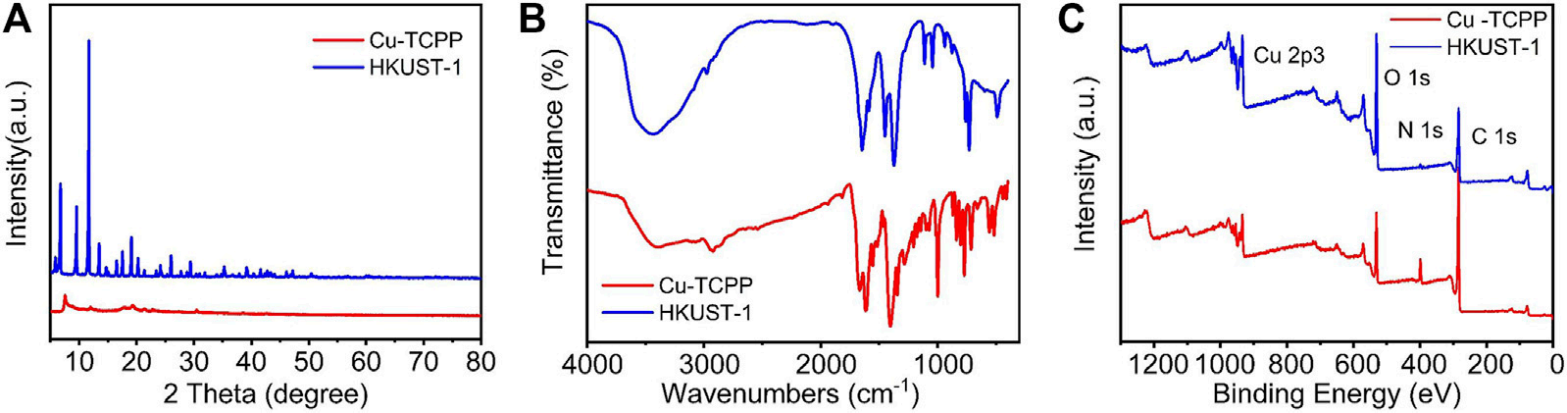
**(A)** XRD pattern of Cu-TCPP and HKUST-1. **(B)** FT-IR pattern of Cu-TCPP and HKUST-1. **(C)** XPS pattern of Cu-TCPP and HKUST-1.

In order to determine the crystal structures of the prepared Cu-MOFs, X-ray diffraction (XRD) was carried out. It can be seen from Figures 2A,D that 2D Cu-MOF exhibited a peak at 2θ = 20° which can be indexed as the (002) crystal plane of Cu-TCPP (Ma et al., 2019). The XRD pattern of HKUST-1 exhibits peaks mainly at the range of 2θ = 5°–20°, corresponding to the previous report, indicating successful synthesis (Wu et al., 2013). It represented a microporous coordination with the cubic crystalline structure. The intense peaks in the XRD demonstrated high crystallinity of the synthesized HKUST-1 samples (Sofi et al., 2019). In addition, the FT-IR spectra were used to identify the functional groups present in the samples. The pattern is shown in Figure 2B. The spectra of 2D Cu-MOF and 3D Cu-MOF presented two strong peaks at around 1,400 and 1,620 cm^−1^, and another strong peak at 3,500 cm^−1^ was contributed by 3D Cu-MOF. The FT-IR spectrum of 3D Cu-MOF demonstrated an almost isobidentate behavior of COO moiety since bands at 1,645, 1,620, 1,570, 1,550, 1,445, and 1,375 cm^−1^ are characteristics of this coordination mode. The latter due to the fact that aniso-bidentate dicopper (II) carboxylate, a type of monomeric clusters, is present in the frameworks (Loera-Serna et al., 2012). Furthermore, the XPS was employed to study the chemical composition and states of Cu-MOFs. The surface characteristics of the synthesized samples were analyzed by XPS. Figure 2C demonstrates a full survey of 2D Cu-MOF and 3D Cu-MOF including Cu 2p3, O 1s, N 1s, and C 1s. In the Cu 2p3 region, the HKUST-1 and Cu-TCPP materials show peaks around 900 eV. These results confirmed that two kinds of Cu-MOFs were prepared successfully (Fan et al., 2019).

### Electrochemical Performances of Modified Electrodes

**FIGURE 3:**
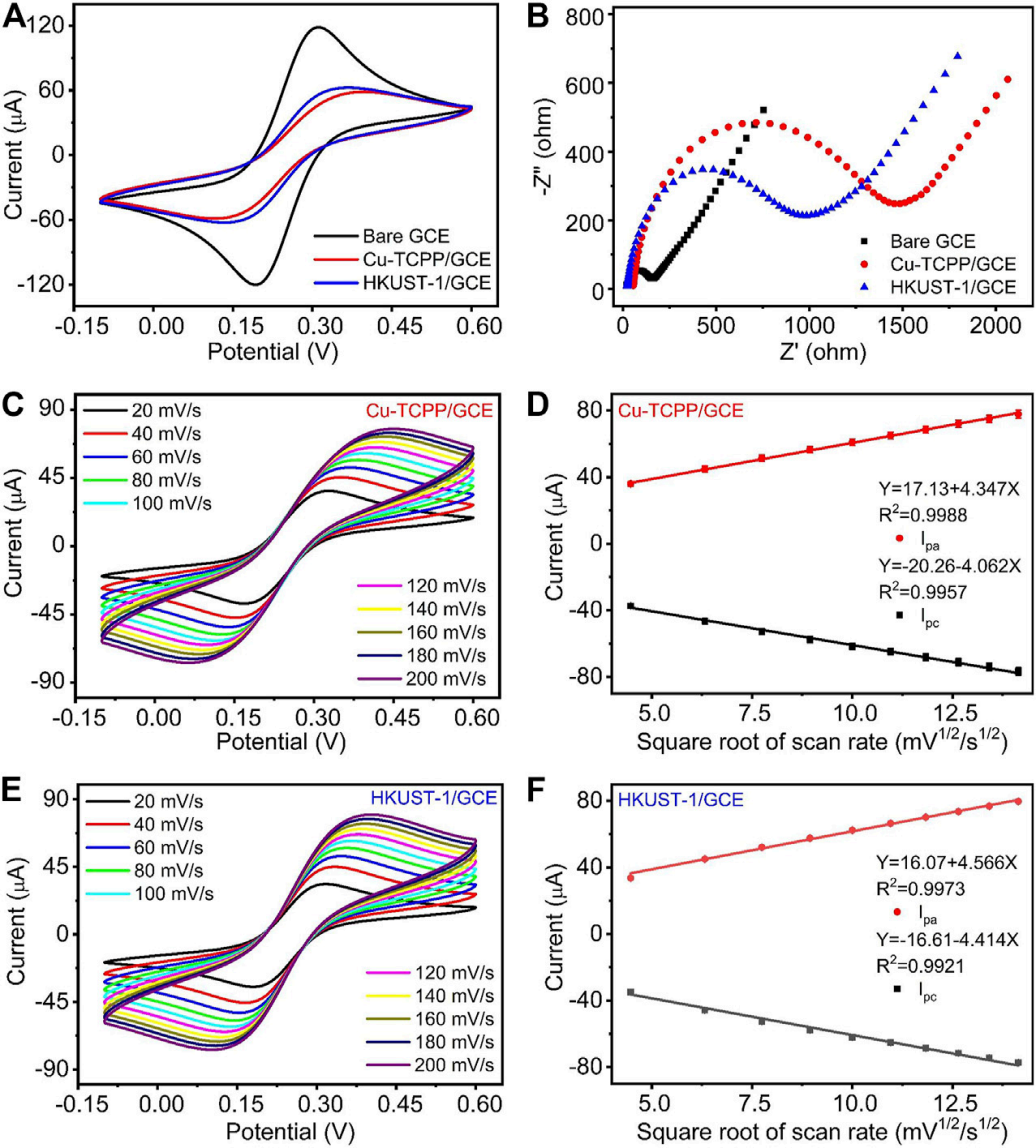
**(A, B)** CV curves and EIS curves of GCE, Cu-TCPP/GCE, and HKUST-1/GCE. **(C)** CV curves of Cu-TCPP/GCE in the aqueous solution containing 5 mM K_3_ [Fe(CN)_6_]/K_4_ [Fe(CN)_6_] and 0.5 M KCl with different scan rates (20, 40, 60, 80, 100, 120, 140, 160, 180, and 200 mV/s). **(D)** The linear relationships between the electrocatalytic peak current with a square root of the scan rate. **(E)** CV curves obtained by HKUST-1/GCE with different scan rates. **(F)** The linear fitting program of the reduction peak current with a square root of the scan rate obtained by HKUST-1/GCE.

To observe the electrochemical performances of bare GCE, Cu-TCPP/GCE, and HKUST-1/GCE, Cyclic voltammetry (CV) and Electrochemical impedance spectroscopy (EIS) were applied to assess their properties. Typically, the EIS plot is composed of a semicircular portion corresponding to the diffusion-limited process and the electron transfer-limited process. The charge transfer resistance (Rct) of the electrode is appropriate to the semicircle diameter. Figures 3A,B are the CV pattern and EIS pattern of different modified electrodes, respectively. Figure 3A illustrates the CV curve of the bare GCE, Cu-TCPP/GCE, and HKUST-1/GCE. Bare GCE demonstrated the highest redox peak current among three kinds of electrodes in the solution of 5 mM K_3_ [Fe (CN)_6_]/K_4_ [Fe(CN)_6_] containing 0.5 M KCl. After coating 6 μL (1 mg/ml) 2D Cu-MOF and 3D Cu-MOF suspension, both the peak current of Cu-TCPP/GCE and HKUST-1/GCE was decreased clearly. The results of EIS measurement matched well with the CV measurement. The EIS diagrams of GCE, Cu-TCPP/GCE, and HKUST-1/GCE are given in Figure 3B. The HKUST-1/GCE had better electrochemical behavior than the Cu-TCPP/GCE with a lower resistance than Cu-TCPP. The Rct value of Cu-TCPP/GCE could reach around 1,500 Ω which is 500 Ω more than the HKUST-1/GCE. Compared with 2D Cu-MOFs, 3D Cu-MOFs exhibit unique chemical and physical properties in electrochemical detection. It could be contributed by the 3D Cu-MOF with a porous structure and rapid icon reaction kinetics to make it possible for fast electron transmission. The Cu2-clusters in HKUST-1 are coordinated via carboxylate groups to form a so-called paddle-wheel unit which makes it possible to access the unsaturated metal sites to boost up the performance in electrochemical sensing (Kim et al., 2012; Cortes-Suarez et al., 2019). All these electrochemical results obtained by EIS and CV measurements have proved that the electrodes modifications were successful.

Furthermore, we studied the influences of scan rates on electrochemical performances. At the range of scan rates from 20 to 200 mV/s, two kinds of modified electrodes exhibited a similar tendency. With the increase of scan rates, the redox current increased as shown in Figures 3C,E. Two kinds of modified electrodes presented a good linear relationship between the reduction peak current and the square root of scan rates as shown in Figures 3D,F. The linear relationship of Cu-TCPP/GCE and HKUST-1/GCE is Y (μA) = -20.26–4.062X (mV^1/2^*s^1/2^), (R^2^ = 0.9957), Y (μA) = -16.61–4.414X (mV^1/2^*s^1/2^), and (R^2^ = 0.9921), respectively. A good linear relationship with the square root of the scan rate indicated fast transfer kinetics and a typical diffusion-controlled electrochemical process.

### Electrochemical Property of Different Modified Electrodes Toward H_2_O_2_

To measure the electrocatalytic activity of the two kinds of different structure Cu-MOFs toward H_2_O_2_ detection, CV measurements were carried out to study the modified electrodes in 0.1 M N_2_ statured PBS solution with or without 10 mM H_2_O_2._ As shown in Supplementary Figure S2, three kinds of electrodes exhibited different current responses to H_2_O_2_. Whether 10 mM H_2_O_2_ was present or not, the bare GCE performed no significant response. Both Cu-TCPP/GCE and HKUST-1/GCE showed an obvious current response, indicating that Cu-MOFs had excellent catalytic performance toward H_2_O_2_ reduction. For comparison, Supplementary Figures S2B,C demonstrate the electrocatalytic activity of different structures of Cu-MOFs under the absence and presence of 10 mM H_2_O_2_. In the 0.1 M N_2_ saturated PBS containing 10 mM H_2_O_2_, the reduction peak current of HKUST-1/GCE could reach nearly 200 μA, which was far beyond the peak current of other two kinds of electrodes. Supplementary Figure S2D is the histogram of the reduction peak current of the electrodes modified by different materials in 0.1 M N_2_ statured PBS with or without 10 mM H_2_O_2_.

**FIGURE 4:**
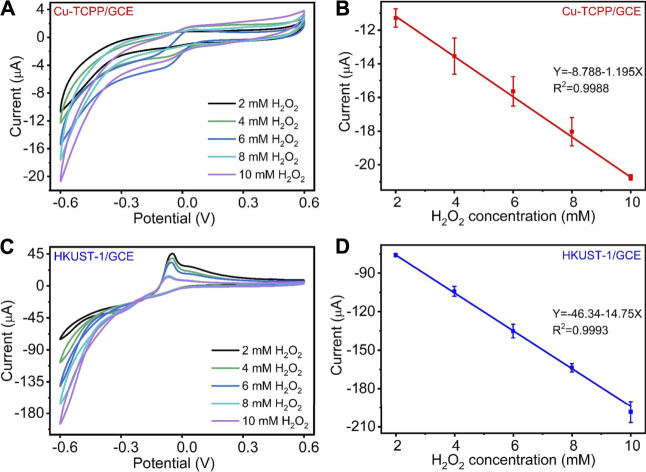
**(A)** CV curves of Cu-TCPP/GCE for different H_2_O_2_ concentrations (2, 4, 6, 8, and 10 mM) with a scan rate of 100 mV/s. **(B)** The linear relationships between the electrochemical peak current and H_2_O_2_ concentration of Cu-TCPP/GCE. **(C)** CV curves of HKUST-1/GCE for different H_2_O_2_ concentrations (2, 4, 6, 8, and 10 mM). **(D)** The linear relationships between the electrochemical peak current and H_2_O_2_ concentration of HKUST-1/GCE.

To further evaluate the Cu-MOF-modified electrodes, we applied a range of concentrations of H_2_O_2_ in 0.1 M N_2_ saturated PBS to measure their electrocatalytic performance as depicted in Figure 4. Figures 4A,C show the CV curves obtained from H_2_O_2_ catalysis by the Cu-MOFs. As displayed in Figures 4A,C, with the H_2_O_2_ concentration increased from 2 to 10 mM, the catalytic reduction current obtained by Cu-TCPP/GCE and HKUST-1/GCE increased significantly. It represented that the prepared electrochemical sensors had a good ability for the H_2_O_2_ electrochemical catalysis. Compared with the peak current of the Cu-TCPP/GCE and HKUST-1/GCE at each H_2_O_2_ level, HKUST-1/GCE had a better electrochemical performance. Furthermore, Cu-TCPP/GCE and HKUST-1/GCE displayed a great linear relationship between the H_2_O_2_ concentration and reduction current. The linear equation of Cu-TCPP/GCE was Y (μA) = -8.788–1.195X (mM) (R^2^ = 0.9988), and the linearity of HKUST-1/GCE was Y (μA) = -46.34–14.75X (mM) (R^2^ = 0.9993) as shown in Figures 4B,D, respectively. Supplementary Figure S3 demonstrates the catalytic reduction currents obtained from two kinds of modified electrodes at different H_2_O_2_ concentrations.

### Amperometric Measurement of H_2_O_2_

In order to assess the applicability of the HKUST-1/GCE for the electrochemical detection of H_2_O_2_, amperometric measurement was used to study the response toward H_2_O_2_ in 0.1 M N_2_ saturated PBS. Applied potential will make a great difference to the current response in electrochemical detection. To investigate the optimum potential toward H_2_O_2_ reduction, I-t curves were obtained by applying different potentials as shown in Supplementary Figure S4. With continuous injection of 0.4 mM H_2_O_2_, the current responses were enhanced with an increasing potential from −0.3 to −0.6 V. Although the HKUST-1/GCE presented the best catalytic activity at the potential of −0.6 V, the background is too high to affect the detection. The potential of −0.3 and -0.4 V could not be selected as the optimal potential because of the low current responses. For these reasons, −0.5 V was chosen as an ideal working potential in the following experiment.

**FIGURE 5:**
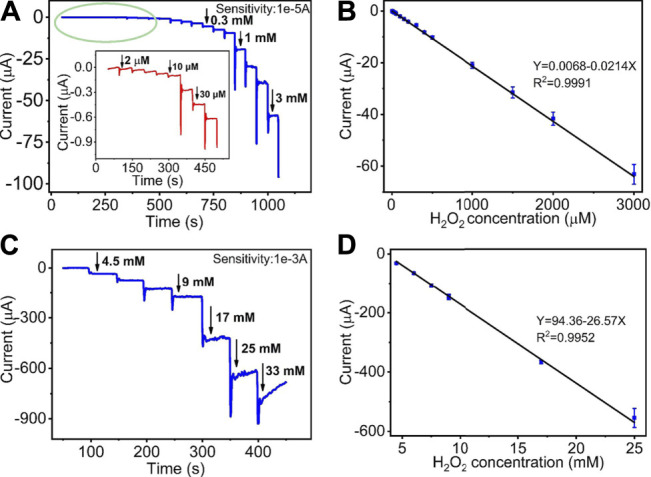
**(A)** I-t curve of HKUST-1/GCE to the successive addition of low concentration of H_2_O_2_ in 0.1 M N_2_ statured PBS at −0.5 V. The inset shows a magnified image of the I-t curve of low concentration from 2 to 40 μM. **(B)** The linear relationship of the response curve of HKUST-1/GCE with different H_2_O_2_ concentrations from 2 μM to 3 mM (R^2^ = 0.9991). **(C)** Amperometric responses of HKUST-1/GCE to the successive addition of high concentration of H_2_O_2_ in 100 mM PBS solution. **(D)** The linear fitting program of the reduction peak current of HKUST-1/GCE with different H_2_O_2_ concentrations from 3 to 25 mM (R^2^ = 0.9952).

Figures 5A,C display the amperometric current response of the quantitative detection of H_2_O_2_ on HKUST-1/GCE. Under the sequential injection of different concentration of H_2_O_2_ to 0.1 M N_2_ saturated PBS with stirring at an ideal potential of −0.5 V, the current responses increased clearly. Figure 5A shows the amperometric I-T curve at the H_2_O_2_ concentrations from 2 μM to 3 mM. The insets of Figure 5A show the amplified image of the current response at the low concentration from 2 to 40 μM. Figure 5C describes the amperometric I-T curve at the H_2_O_2_ concentrations from 3 to 25 mM. Furthermore, the current responses increased and reached a stable state within 10 s after each step of H_2_O_2_ injection, indicating the rapid response of HKUST-1/GCE in the electrochemical detection of H_2_O_2._ Figures 5B,D illustrate a great linear relationship between concentrations and the current response. The linear regression equation was Y (μA) = 0.0068–0.0214X (μM) in the H_2_O_2_ concentrations of 2 μM–3 mM with a correlation coefficient of 0.9991. Good linearity (from 3 to 25 mM) was Y (μA) = 94.36–26.57X (mM) (R^2^ = 0.9952). The LOD was found as 0.68 μM with a signal-to-noise ratio of 3. The comparison of the modified electrodes for the detection of H_2_O_2_ in previous reports is given in Table 1. Compared with other research, HKUST-1/GCE exhibited good electrochemical catalysis to H_2_O_2_ reduction with an extended linear range and a lower LOD. The results could be attributed to the 3D porous structures and fast electron transmission of the materials. All these synergistic factors ensured the excellent electrocatalytic performance of the HKUST-1/GCE.

**TABLE 1:** Comparison of different electrochemical platforms for hydrogen peroxide sensing.

Electrodes	Detection potential (V)	Linear range (μM)	LOD (μM)	References
NC@rGO^a^	−0.4 V (vs. Ag/AgCl)	5–20,000	3.3	Li et al. (2018b)
Ag@ZIF-67/GCE^b^	−0.25 V (vs. SCE)	5–275; 775–2,775	1.5	Dong et al. (2019)
		4,775–16,775		
C-ZIF-67/GCE	−0.35 V (vs. SCE)	2.5–212.5	0.7	Dong and Zheng, (2020)
		212.5–1662.5		
		1662.5–6662.5		
HPB/CS/GCE^c^	0.1 V (vs. SCE)	8–1848	2.6	Sheng et al. (2017)
CuCo_2_O_4_	−0.55 V (vs. Ag/AgCl)	10–8900	3.0	Cheng et al. (2020)
Cu-MOF	−0.2 V (vs. Ag/AgCl)	1–900	1.0	Zhang et al. (2015)
IE-MoS_2_(3.0)^d^	−0.65 V (vs. Ag/AgCl)	0.23–2,200	0.2	Shu et al. (2019)
		2,200–14220		
Cu-MOF/ERGO/ITO^e^	−0.3 V (vs. Ag/AgCl)	4–17,334	0.44	Golsheikh et al. (2020)
HKUST-1/GCE	−0.5 V (vs. Ag/AgCl)	2–3000	0.68	This work
		3000–25,000		

a NC, nitrogen-rich core-shell; rGO, reduced graphene oxide.

b ZIF, zeolitic imidazolate frameworks.

c HPB, hollow Prussian blue; CS, chitosan.

d IE, interlayer-expanded.

e ERGO, electrochemically reduced graphene oxide; ITO, indium tin oxide.

### Selectivity and Stability of HKUST-1/GCE

**FIGURE 6:**
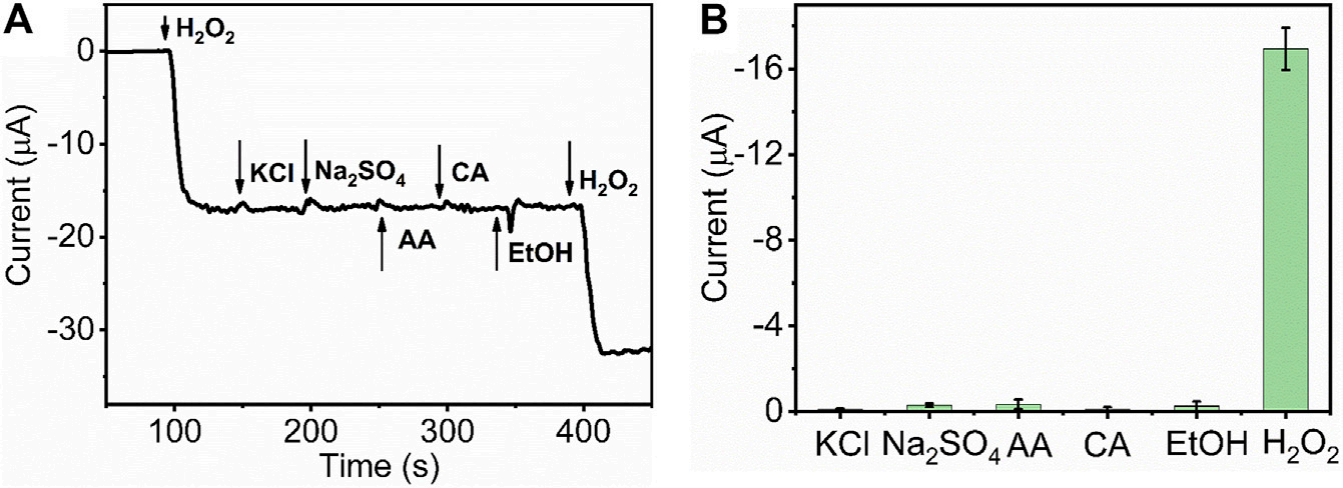
**(A)** I-t curve of HKUST-1/GCE with the successive injection of 1 mM H_2_O_2_, 10 mM KCl, 10 mM Na_2_SO_4_, 10 mM CA, 10 mM AA, C_2_H_5_OH, and 1 mM H_2_O_2_ in 0.1 M N_2_ statured PBS at an applied potential of −0.5 V. **(B)** Histogram of the current peak obtained by HKUST-1/GCE of H_2_O_2_ and other potential interferences.

The selectivity of the sensor represents the ability of real sample detection and practicability. To investigate the catalytic specificity of HKUST-1/GCE further, amperometric measurement was used to study the anti-interference capability of HKUST-1/GCE. At the operating potential of −0.5 V, 1 mM H_2_O_2_,10 mM potassium chloride (KCl), 10 mM sodium sulfate (Na_2_SO_4_), 10 mM ascorbic acid (AA), 10 mM citric acid (CA), ethanol absolute, and 1 mM H_2_O_2_ were injected in 10 ml 0.1 M N_2_ statured PBS successively. Figure 6A displays the I-T curve obtained by the catalysis of H_2_O_2_ and some potential interferences. The obvious and rapid current response occurred when the 1 mM H_2_O_2_ was injected into the PBS. In contrast, no obvious current change could be observed after ten folds of interfering species injection in the same solution. Figure 6B displays the current response change of the H_2_O_2_ and other potential interferences. All these results indicated the HKUST-1/GCE sensor with high selectivity for the electrochemical detection of H_2_O_2_ in the presence of common interferences.

In addition, we studied the stability of the HKUST-1/GCE electrochemical sensors using CV measurement in the PBS solution containing 10 mM H_2_O_2_ at the same condition. The results of the stability of the electrochemical sensor are displayed in Supplementary Figure S5. The electrochemical current responses of the sensors retained 90% of their initial value after 5 days. The result of the experiment indicated the good stability of the 3D Cu-MOF-modified electrodes.

### Real Sample Analysis of Dairy Products

Generally, H_2_O_2_ is used as an additive in the food industry for storage, stability, and other purposes. However, over content of H_2_O_2_ will have a side effect on human beings by causing many diseases. Thus, there is great importance for rapid and specific detection of H_2_O_2_ in milk samples using a convenient method. Milk samples were purchased from a local supermarket. The practical application of the prepared sensor was carried out to measure the concentration of H_2_O_2_ in milk samples. The standard addition method and amperometric measurements were used in this experiment section. The milk samples were diluted 20 times using 0.1 M N_2_ saturated PBS (pH 7.2). A range of concentrations of H_2_O_2_ (0, 40, 80, and 120 μM) were added to the milk sample, respectively. Then, milk samples containing different concentrations of H_2_O_2_ were ready for analysis. The I-T curve obtained by the amperometric measurements was presented in Supplementary Figure S6. As shown in Supplementary Figure S6, no obvious amperometric current response could be seen at the first injection of the diluted milk sample without additional H_2_O_2_. It proves that the milk sample does not contain endogenous H_2_O_2_. With the subsequent injection of milk samples containing different concentrations of additional H_2_O_2_, the current response increased rapidly and obviously, indicating that the sensor is suitable for H_2_O_2_ detection with good adaptability and practicality in a complex aqueous system. Furthermore, the standard addition method was carried out to calculate the relative standard deviation (RSD) and the recovery rate based on the previous linear regression equation. As shown in Table 2, the RSD was less than 8%, and the average recovery rate was 100.2%, 97.1%, and 96.1% (n = 3), respectively. These results demonstrated that the prepared sensor is highly reproducible and effective for H_2_O_2_ sensing in milk samples.

**TABLE 2:** Detection of H_2_O_2_ in the milk sample using HKUST-1/GCE (*n* = 3).

Sample	Added (μM)	Average founded (μM)	Average recovery (%)	RSD (%)
Milk	40.0	40.1	100.2	4.6
	80.0	77.7	97.1	6.1
	120.0	115.4	96.1	7.7

## Conclusion

In summary, two kinds of pristine Cu-MOFs with different structures were synthesized successfully for the comparison of morphology and electrocatalytic ability. 3D Cu-MOFs with an octahedral structure performed lower resistance and higher current peak response for the electrochemical catalysis of H_2_O_2_ than 2D Cu-MOF, demonstrating that the morphology of the Cu-MOFs could influence the electrochemical performance in H_2_O_2_ reduction. The HKUST-1/GCE presented two wide linear ranges (2 μM–3 mM and 3–25 mM) and a low detection limit of 0.68 μM for H_2_O_2_ detection in 0.1 M N_2_ saturated PBS. Furthermore, the prepared sensor had been applied for the detection of H_2_O_2_ in milk samples, showing its satisfactory practicability and prospect. This work provided an idea and strategy for the electrochemical detection of H_2_O_2_. This sensor had great potential for electrochemical detection in the food industry and agricultural system to meet the demand of rapid detection and selectivity in analyses.

## Data Availability

The original contributions presented in the study are included in the article/Supplementary Material; further inquiries can be directed to the corresponding authors.
