# Supplementary material for: 2D/3D Copper-Based Metal-Organic Frameworks for Electrochemical Detection of Hydrogen Peroxide
Source: Front Chem. 2021 Oct 7;9:743637. doi: 10.3389/fchem.2021.743637 (PMC8530376; doi:10.3389/fchem.2021.743637)
Supplement: Supplementary file 1 [file DataSheet1.docx]

**Supplementary Material**

**2D/3D Copper-Based Metal-Organic Frameworks for Electrochemical Detection of Hydrogen Peroxide**

Xiangjian Guo^1,2^, Chuyan Lin^1^, Minjun Zhang^1^, Xuewei Duan^1^, Xiangru Dong^2^,Duanping Sun^2^*, Jianbin Pan^3^*, Tianhui You^1^*

^1^ School of Nursing, Guangdong Provincial Key Laboratory of Pharmaceutical Bioactive Substances, Guangdong Pharmaceutical University, Guangzhou 510006, Guangdong, China

^2^ Center for Drug Research and Development, Guangdong Provincial Key Laboratory of Pharmaceutical Bioactive Substances, Guangdong Pharmaceutical University, Guangzhou 510006, China

^3^ State Key Laboratory of Analytical Chemistry for Life Science, School of Chemistry and Chemical Engineering, Nanjing University, Nanjing 210023, China

* Correspondence:

Duanping. Sun, Jianbin. Pan, Tianhui. You.

sundp@gdpu.edu.cn, jbpan@nju.edu.cn, youth888cn@aliyun.com


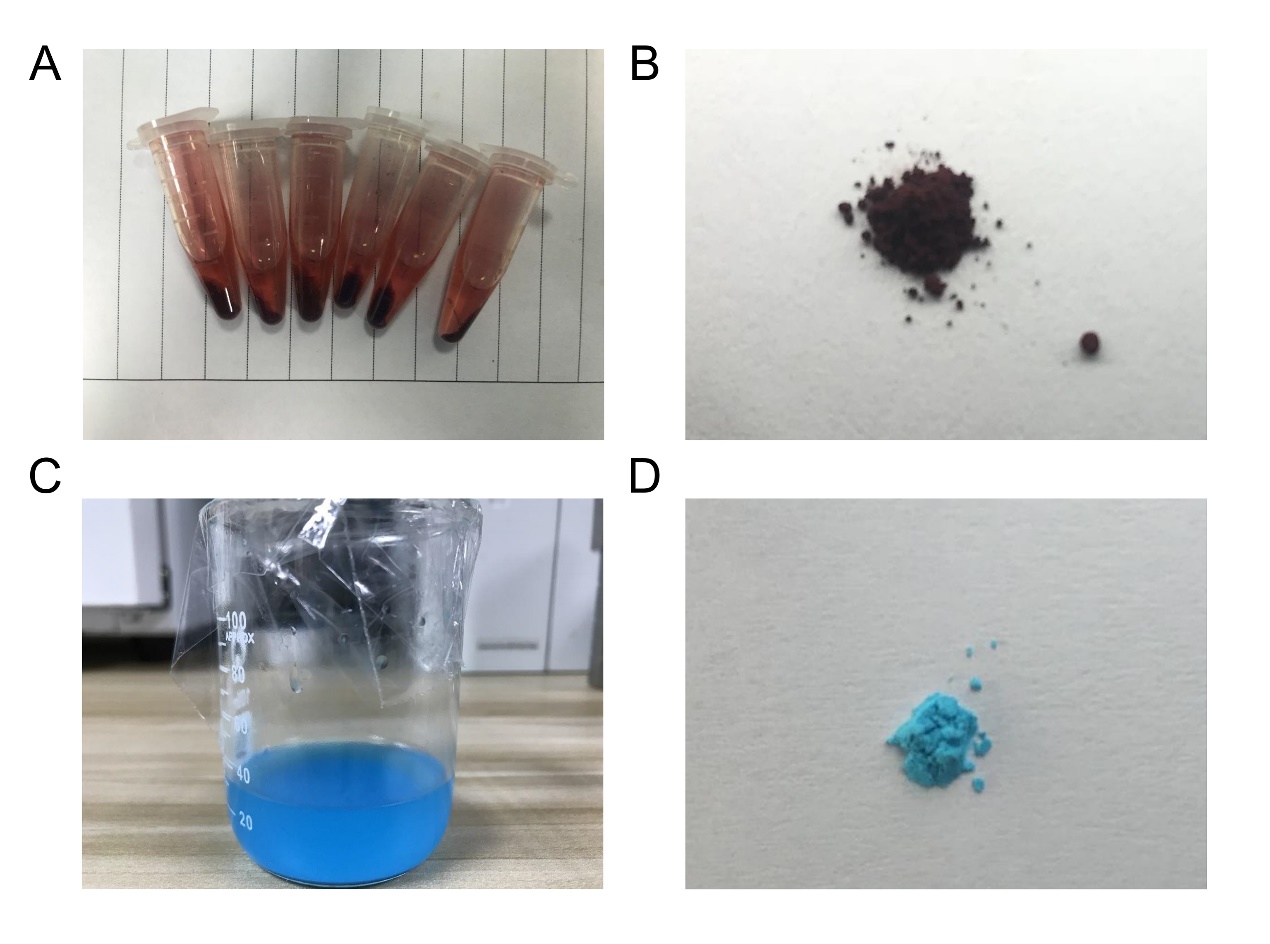


**Figure S1.** (A, B) Synthesis of Cu-TCPP. (C, D) Synthesis of HKUST-1.


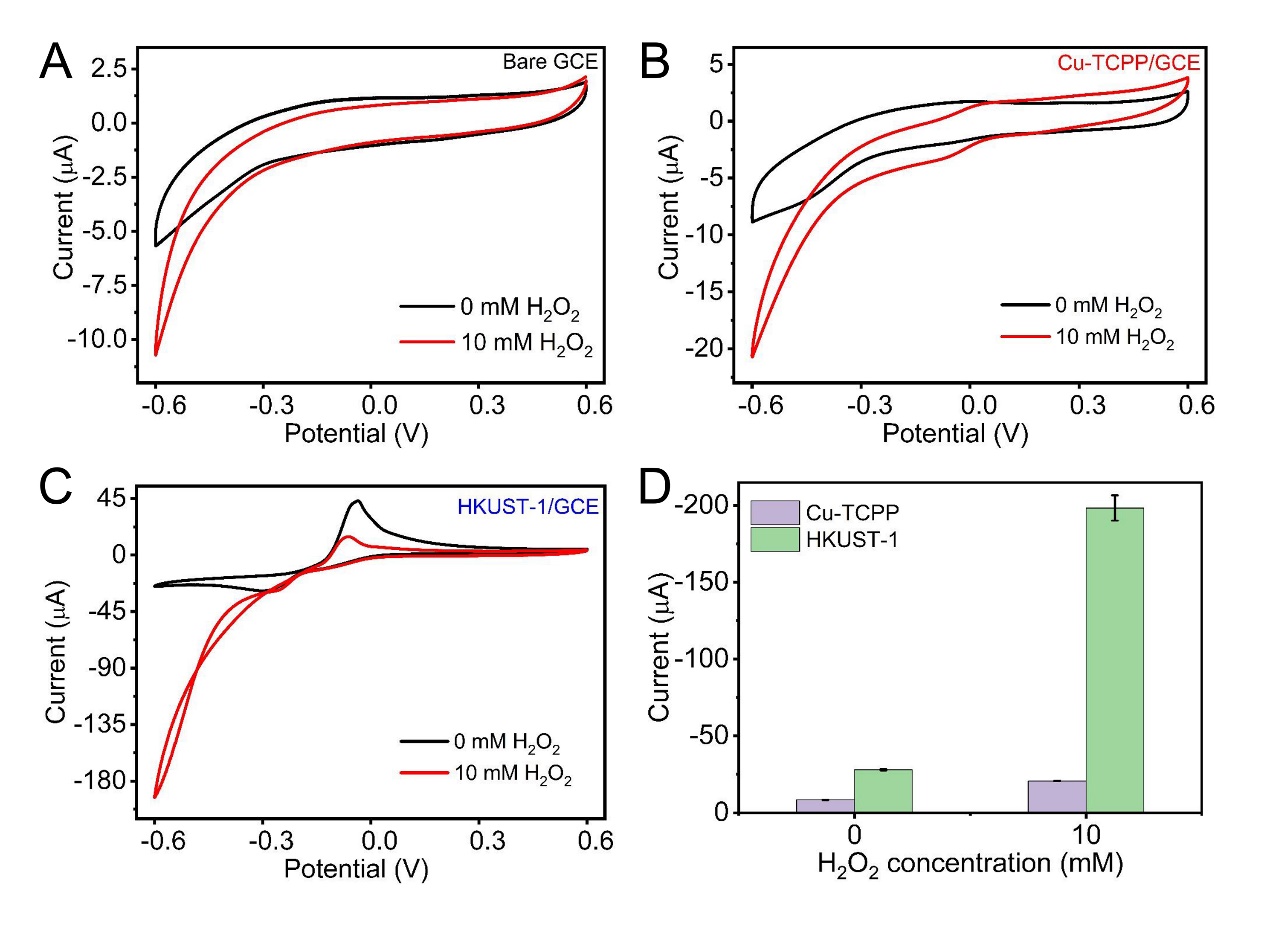


**Figure S2.** (A,B,C) CV curves of GCE, Cu-TCPP/GCE, HKUST-1/GCE in 0.1 M PBS (the black curve) and 10 mM H_2_O_2_ (the red curve). (D) Comparison of the current peak of Cu-TCPP/GCE and HKUST-1/GCE in 0.1 M PBS with absent or present 10 mM H_2_O_2_.(n=3)


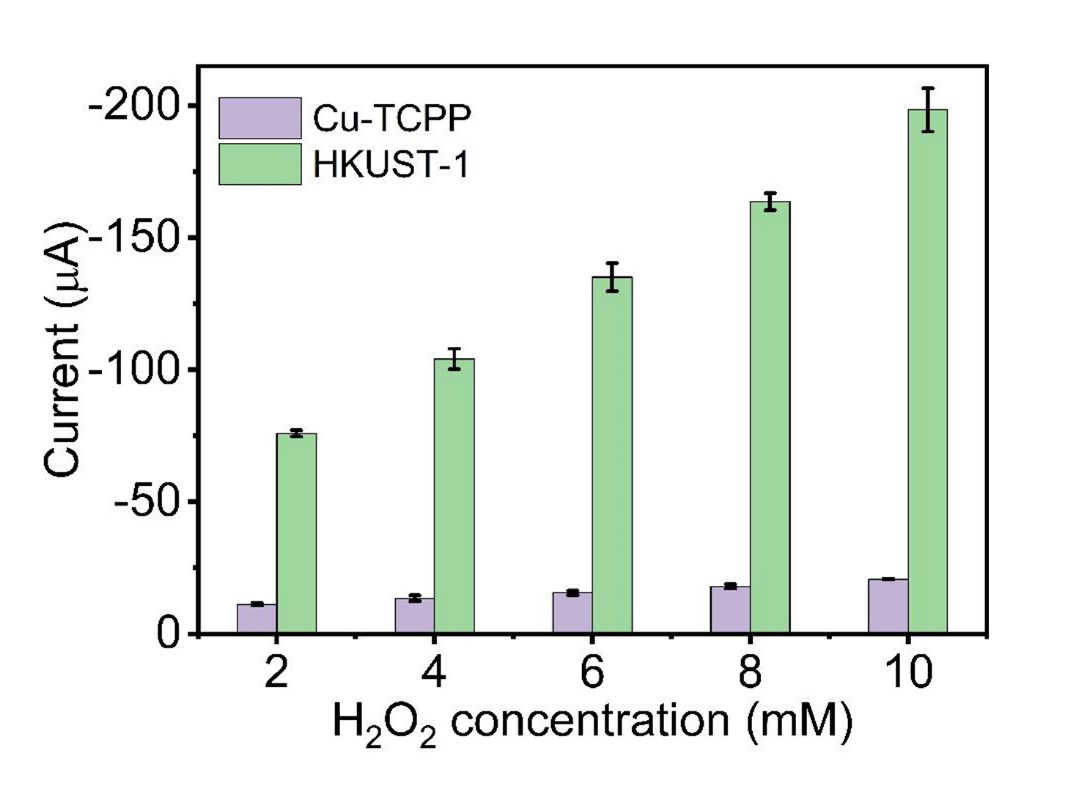


**Figure S3.** Histograms of the current peak of Cu-TCPP/GCE and HKUST-1/GCE in 0.1 M PBS at different H_2_O_2_ concentrations (2, 4, 6, 8, and 10 mM) (n=3).


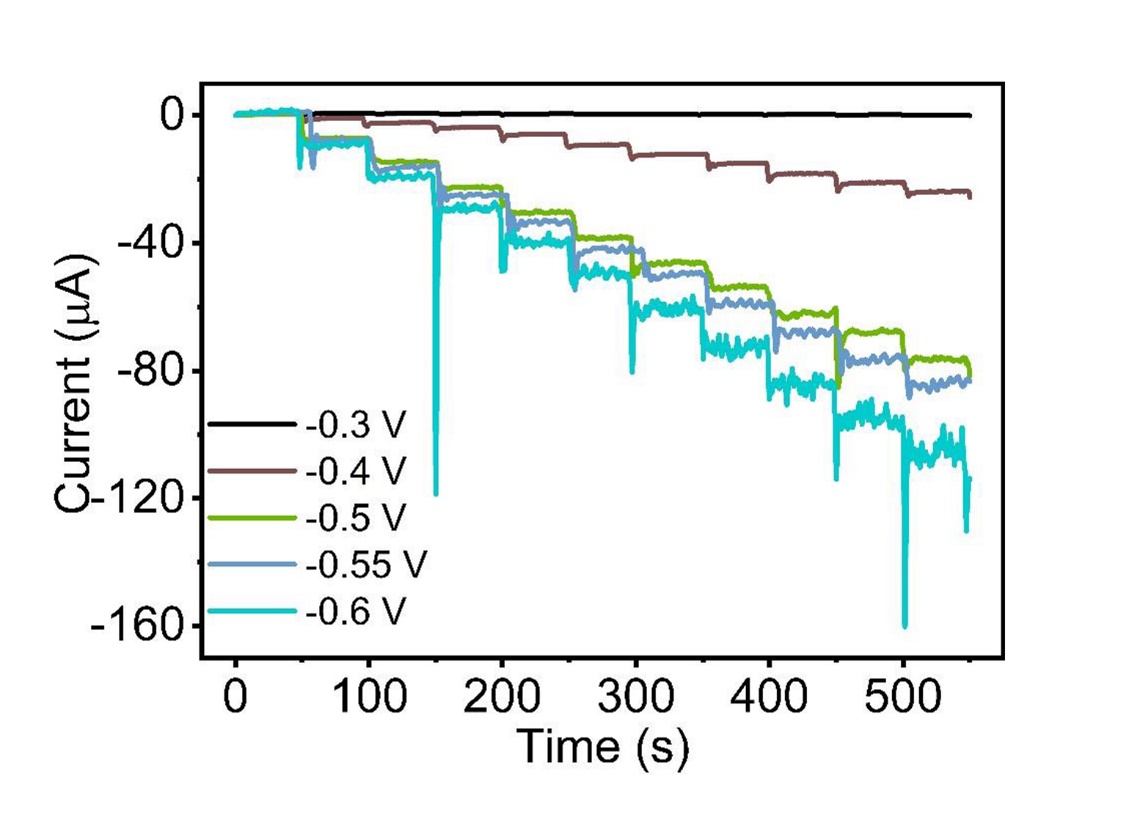


**Figure S4.** Amperometric responses of HKUST-1/GCE with different applied potentials (-0.3, -0.4, -0.5, -0.55, -0.6 V) by successively injecting 0.4 mM H_2_O_2_ in PBS solution.


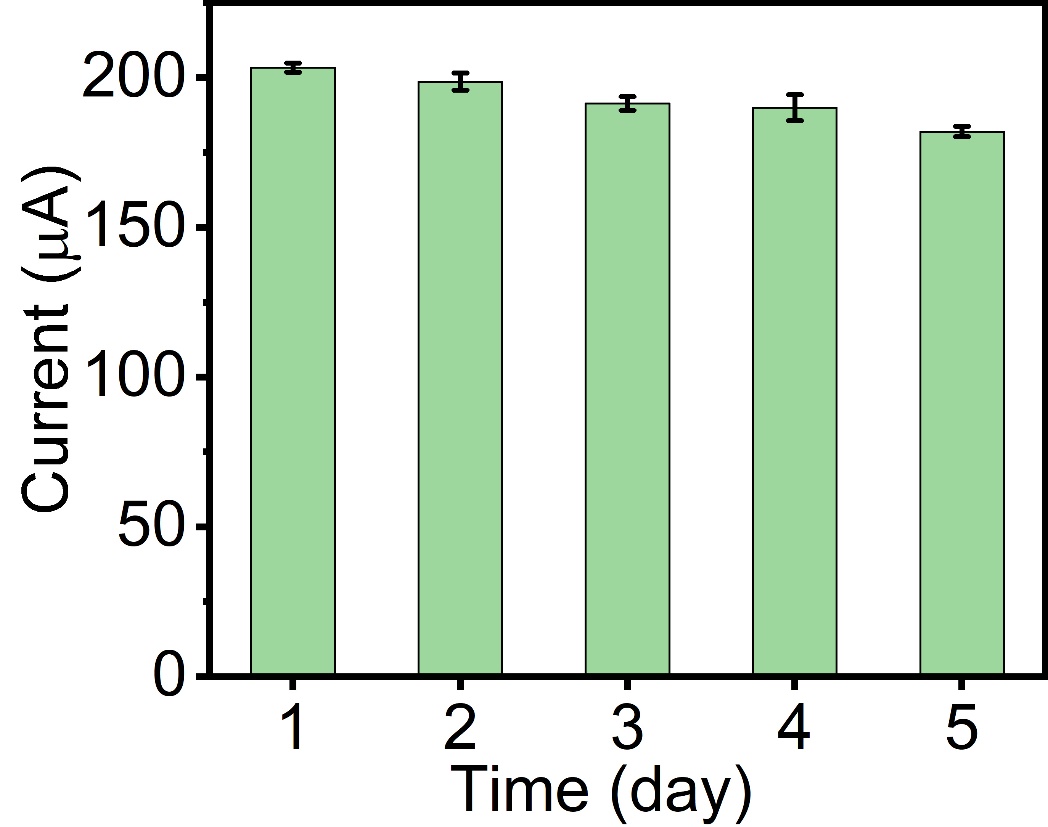


**Figure S5.** Histograms of stability test over 5 day by CV measurements in PBS solution containing 10 mM H_2_O_2_ at a scan rate of 100 mV·s^-1^.


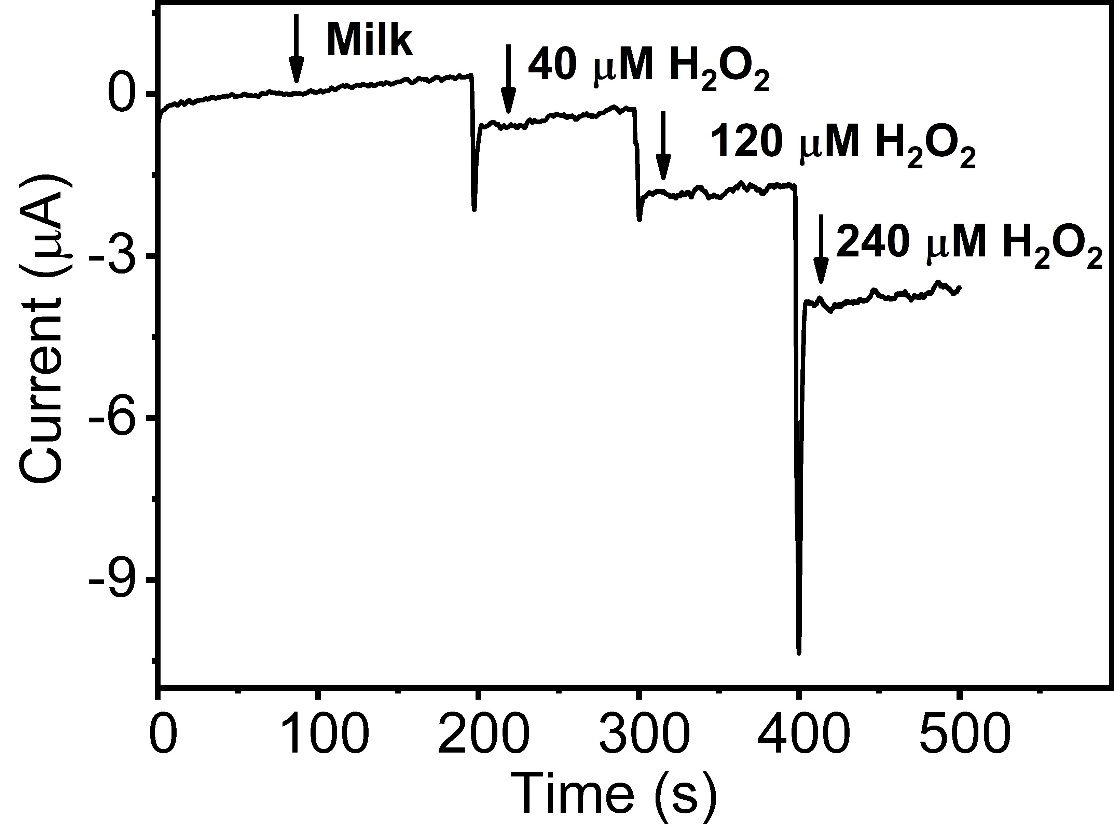


**Figure S6.** Amperometric responses of HKUST-1/GCE to the successive addition of milk sample containing different concentration of H_2_O_2_ (0, 40, 80, 120 μM) in PBS solution at an applied potential of -0.5 V.
